# Transcriptomic Comparison of Rice lncRNAs in Response to Feeding by Brown Planthopper Populations with Different Virulence

**DOI:** 10.3390/ijms26083486

**Published:** 2025-04-08

**Authors:** Yaxuan Wang, Xinfeng Wang, Kunjie Zhang, Jing Xiao, Fang Liu, Houhong Yang, Yubiao Cai, Fengxiang Lai, Qiang Fu, Pinjun Wan

**Affiliations:** The National Key Laboratory of Rice Biological Breeding, China National Rice Research Institute, Hangzhou 311401, China; 82101232346@caas.cn (Y.W.); wangxinfeng0211@163.com (X.W.); 13396545151@163.com (K.Z.); xiaojing00004@163.com (J.X.); 18453066791@163.com (F.L.); 82101221215@caas.cn (H.Y.); caiyubiao2020@163.com (Y.C.); laifengxiang@caas.cn (F.L.); fuqiang@caas.cn (Q.F.)

**Keywords:** strand-specific lncRNA-seq, jasmonic acid, brown planthopper and rice interaction, virulence

## Abstract

The brown planthopper (BPH) is one of the major rice pests causing significant damage to rice production worldwide, due to its high reproductive capacity and strong migratory ability. A series of BPH-resistant rice varieties have been developed, but the durability of effective resistance is constrained by the evolution of BPH virulence, requiring in-depth insights into resistance mechanisms. In this paper, we used strand-specific lncRNA-seq to characterize the lncRNA regulatory mechanisms on rice response to BPH infestation. Overall, 4321 lncRNAs were identified, 60 of which were significantly upregulated in response to BPH infestation, specifically differing between BPH populations with variable virulence. Differential expression analysis and qRT-PCR validation showed that these lncRNAs are involved in the regulation of several defense pathways, including jasmonic acid signaling and flavonoid biosynthesis, with their distinct roles in resistant and susceptible rice varieties. Notably, lncRNAs like *LNC_002533* were found to be negatively correlated with flavonoid biosynthesis, suggesting a potential role in modulating rice defense responses. In contrast, *LNC_001986* and *LNC_000397* were positively correlated with genes involved in glutathione metabolism, which may be associated with enhanced resistance. These findings highlight the critical regulatory functions of lncRNAs in rice-BPH interactions and provide a molecular framework for improving rice resistance through targeted genetic engineering. This study significantly contributes to functional genomics by elucidating lncRNA-mediated regulatory mechanisms and offers promising avenues for developing durable pest-resistant rice varieties.

## 1. Introduction

Rice (*Oryza sativa*) is a staple crop worldwide and feeds over half of the global population. A stable and high rice yield is important for global food security. Nonetheless, different biotic stresses are an ongoing threat to rice production, of which the brown planthopper (BPH, *Nilaparvata lugens*) is among the most destructive [1]. BPH is a notorious pest that damages rice plants by direct feeding and by transmitting plant viruses [2]. The high reproductive capacity, migratory behavior, and evolution of virulence against resistant rice varieties of this pest is making pest management and sustainable rice production difficult [3]. In addition, the economic losses associated with BPH infestations have long-term effects on smallholder farmers in rice-dependent areas, making it critical to address this issue [4].

To combat BPH, numerous resistant rice varieties have been developed by utilizing genetic loci such as *Bph3*, which has shown broad-spectrum and durable resistance in certain rice varieties [5]. The *Bph3* locus, consisting of three *lectin receptor-like kinase* genes (*OsLecRK1-OsLecRK3*), effectively confers resistance to multiple BPH biotypes, which are specific populations categorized based on their virulence against different resistance genes [6]. However, the effectiveness of resistant varieties often diminishes over time due to the adaptation and evolution of virulence in BPH populations. For instance, through prolonged forced feeding, we successfully established a new BPH population capable of overcoming the resistance of IR56 rice (harboring the *Bph3* resistance gene). This newly formed population, designated as IR56-BPH, demonstrated the ability to infest and damage IR56 rice, confirming its evolved virulence [7]. Despite significant progress in identifying resistance genes and breeding resistant rice varieties, the regulatory networks and molecular pathways governing rice responses to BPH infestation remain inadequately understood.

Long noncoding RNAs (lncRNAs) are RNA transcripts longer than 200 nucleotides that lack protein-coding potential and have recently emerged as key regulators of various biological processes [8,9]. In plants, lncRNAs play a crucial role in regulating growth, development, and responses to both abiotic and biotic stresses [10]. These molecules exert their regulatory effects through mechanisms like chromatin remodeling, transcriptional and post-transcriptional control, and by acting as decoys or sponges for microRNAs [11]. For example, the lncRNA *ELENA1* in *Arabidopsis* positively regulates immunity against *Pseudomonas syringae* by interacting with RNA-binding proteins to modulate the expression of pathogenesis-related genes [12,13]. Similarly, in tomatoes, *lncRNA16397* enhances resistance to *Phytophthora infestans* by promoting the expression of its adjacent gene *SlGRX*, which helps reduce reactive oxygen species (ROS) accumulation and alleviates cell membrane damage [14]. In rice, the lncRNA *ALEX1* contributes to enhanced resistance against *Xanthomonas oryzae* pv. *Oryzae* (*Xoo*) by upregulating jasmonic acid (JA)-responsive genes [15]. Furthermore, *lncRNA13262*, *lncRNA01308*, and *lncRNA23468* have been identified as *miR482b* sponges, involved in pathogen defense. Notably, the overexpression of *lncRNA23468* inhibits *miR482b* activity, significantly upregulating *nucleotide binding site plus leucine rich repeat* (*NBS-LRR*) gene expression and enhancing immunity against *P. infestans* [16]. Conversely, the tomato lncRNA *lncRNA39896* interacts with miR166b to negatively regulate insect resistance in tomatoes [17].

These findings underscore the important roles of lncRNAs in plant immunity, particularly in mediating responses to microbial pathogens and insect pests. However, most functional studies of plant lncRNAs have focused on model organisms or bacterial pathogens, while the regulatory roles of lncRNAs in rice responses to insect herbivory, especially in the context of BPH populations with different virulence, remain largely unexplored. Addressing this gap is crucial for advancing our understanding of host–insect interactions and for developing durable pest-resistant rice varieties. Therefore, this study aims to elucidate the regulatory roles of lncRNAs in rice defense against BPH populations of varying virulence, with a focus on their effects on JA signaling and secondary metabolite pathways. Using strand-specific lncRNA-seq and comparative transcriptomic analysis, we identified lncRNAs in IR56 rice plants infested by BPH populations with differing virulence levels. By comparing expression profiles between rice infested with a highly virulent BPH population (IR56-BPH) and a low-virulence one (TN1-BPH), we identified a set of differentially expressed lncRNAs (DElncRNAs) potentially associated with resistance or susceptibility. Functional enrichment analysis and lncRNA-mRNA coexpression networks indicated that these DElncRNAs are involved in key defense pathways, including JA signaling and flavonoid biosynthesis. qRT-PCR validation confirmed the expression patterns of several lncRNAs and their predicted target genes. For instance, the expression of *LNC_002533* was negatively correlated with that of flavonoid biosynthesis-related genes such as *OsLAR*, suggesting a potential role in downregulating flavonoid biosynthesis-mediated defense responses, though direct regulatory interactions require further validation. Conversely, *LNC_001986* and *LNC_000397* was positively correlated with genes involved in glutathione metabolism, implying a potential role in enhancing resistance under TN1-BPH infestation. These findings provide new insights into the regulatory functions of lncRNAs in rice-insect interactions and identify promising molecular markers or targets for breeding rice varieties with durable, broad-spectrum resistance to BPH.

## 2. Results

### 2.1. Genome-Wide Identification of Rice lncRNAs Responsive to BPH Infestation

To comprehensively characterize the expression landscape of lncRNAs during rice–BPH interaction, IR56 rice seedlings were infested with two BPH populations differing in virulence: IR56-BPH and TN1-BPH. Stem tissues were harvested at 24 h post-infestation for RNA extraction. Three strand-specific lncRNA-seq libraries were constructed: non-infested control (IR56-CK), IR56-BPH-infested (IR56-IR), and TN1-BPH-infested (IR56-TN). Sequencing yielded a total of 283,942,306 clean reads, of which 86.57% were successfully mapped to the reference rice genome (Table S1).

**Figure 1:**
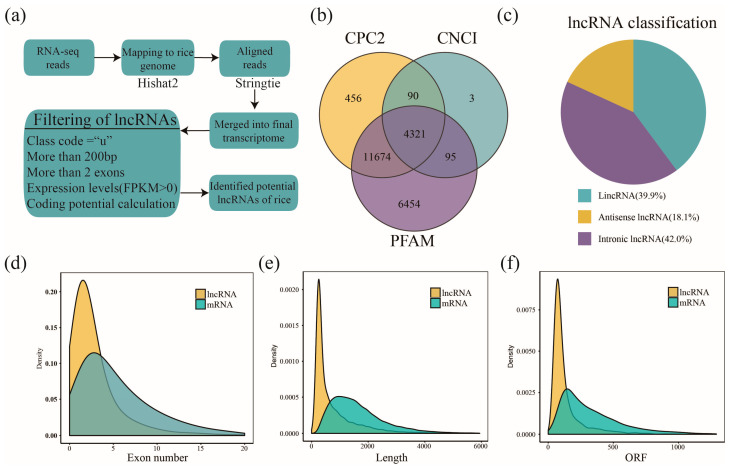
Identification and classification of lncRNAs in rice under brown planthopper stress. (**a**) Workflow for the identification of long noncoding RNAs (lncRNAs) in rice. RNA-seq reads were aligned to the rice genome using HISAT2 and assembled into transcripts using StringTie. Candidate lncRNAs were filtered based on transcript class code (“u”), length (>200 bp), exon number (>2), expression levels (FPKM > 0), and coding potential calculation using CPC2, CNCI, and PFAM tools. (**b**) Venn diagram showing the intersection of candidate lncRNAs predicted by CPC2, CNCI, and PFAM analyses, identifying a total of 4321 lncRNAs. (**c**) Classification of lncRNAs into three categories based on their genomic locations: intergenic lncRNAs (39.9%), antisense lncRNAs (18.1%), and intronic lncRNAs (42.0%). (**d**) Distribution of exon numbers in lncRNAs and mRNAs. lncRNAs tend to have fewer exons compared to mRNAs. (**e**) Length distribution of lncRNAs and mRNAs, with lncRNAs generally being shorter than mRNAs. (**f**) Open reading frame (ORF) length distribution of lncRNAs and mRNAs.

LncRNA candidates were identified using a stringent pipeline (Figure 1a). Transcripts were aligned to the rice genome using HISAT2 (v2.1.0) and assembled using StringTie (v1.3.5). Candidate lncRNAs were filtered based on transcript class code (“u”), length (>200 bp), expression levels (FPKM > 0), and coding potential calculated using the Coding Potential Calculator (CPC), Coding-Non-Coding Index (CNCI), and Pfam analysis. Transcripts longer than 200 nucleotides and containing at least one exon were selected as lncRNA candidates. While multi-exon transcripts were prioritized during filtering, we retained high-confidence single-exon lncRNAs that passed all coding potential filters (CPC2, CNCI, and Pfam) and exhibited conserved genomic features characteristic of functional lncRNAs. A total of 4321 high-confidence lncRNAs were identified based on transcript length (>200 nt), exon number (>2), and coding potential, as assessed using the CPC2, CNCI, and Pfam databases (Figure 1b).

These lncRNAs were classified into three categories based on genomic locations: intergenic lncRNAs (lincRNAs), intronic lncRNAs, and antisense lncRNAs [18]. Among them, intronic lncRNAs were the most abundant (42.0%), followed by lincRNAs (39.9%) and sense-antisense lncRNAs (18.1%) (Figure 1c). To better understand the potential biological functions of these lncRNAs, we examined the significance of their genomic classifications. Each category of lncRNA has distinct biological significance [19]. For instance, lincRNAs, which are transcribed from intergenic regions, often function as independent transcriptional units and can regulate gene expression through chromatin remodeling or by acting as molecular scaffolds. Intronic lncRNAs, derived from introns of protein-coding genes, may play roles in splicing regulation or modulate the expression of their host genes. Antisense lncRNAs, which overlap with coding genes on the opposite strand, can influence gene expression by interfering with mRNA stability or translation. A comprehensive list of identified lncRNAs and their classifications is provided in Supplementary Table S2.

To compare their genomic features with mRNAs, we analyzed transcript structure and found that lncRNAs had fewer exons and shorter transcript lengths than mRNA (Figure 1d,e, Tables S2 and S3). Specifically, 43.30% (1871 of 4321) of lncRNAs were single-exon, while only 14.02% (12,318 of 87,836) of mRNAs were single-exon (Figure 1d). Additionally, 42.28% (1827 of 4321) of the lncRNAs exceeded 500 nucleotides in length, compared to 94.46% (82,972 of 87,836) of mRNAs (Figure 1e). To further support the classification of the identified transcripts as non-coding, we compared the length of their predicted open reading frames (ORFs) with those of mRNAs. As expected, the average ORF length of lncRNAs was significantly shorter than that of mRNAs (Figure 1f), which is consistent with their limited protein-coding potential. This pattern aligns with previous findings and supports the non-coding nature of these transcripts. These distinct genomic characteristics support the classification and noncoding nature of the identified lncRNAs and provide a robust foundation for downstream expression and functional analyses.

### 2.2. Identification of lncRNAs Related to BPH Resistance

**Figure 2:**
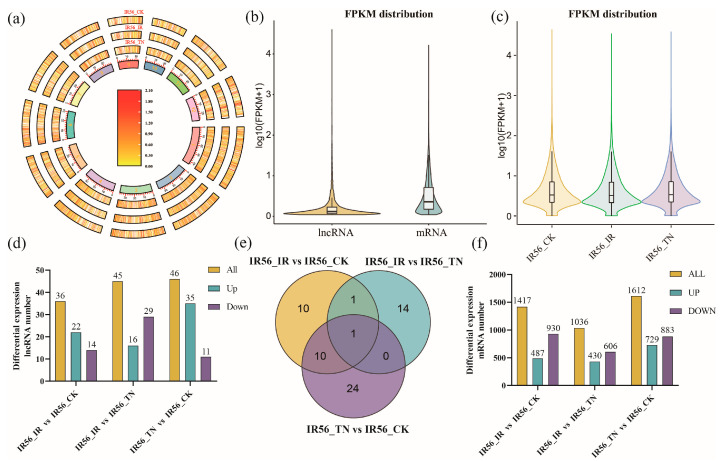
Expression profiles and differential analysis of lncRNAs and mRNAs in rice under brown planthopper stress. (**a**) Genome-wide expression distribution of lncRNAs across the 12 rice chromosomes (Chr1-Chr12) in three groups: IR56-CK, IR56-IR, and IR56-TN. The heatmap shows the normalized expression levels (log_10_(FPKM + 1)) of lncRNAs. (**b**) Violin plot comparing the overall expression levels (log_10_(FPKM + 1)) of lncRNAs and mRNAs. lncRNAs exhibit lower expression levels compared to mRNAs. (**c**) Violin plots showing the expression distribution (log_10_(FPKM + 1)) of lncRNAs across the three groups: IR56-CK, IR56-IR, and IR56-TN. (**d**) Bar chart depicting the number of differentially expressed lncRNAs (DElncRNAs) across comparisons: IR56-IR vs. IR56-CK, IR56-TN vs. IR56-CK, and IR56-IR vs. IR56-TN. DElncRNAs are categorized into upregulated and downregulated groups. (**e**) Venn diagram showing the overlap of upregulated DElncRNAs among the three comparisons. (**f**) Bar chart showing the number of differentially expressed mRNAs across the same comparisons.

The chromosomal distribution of all identified lncRNAs was mapped across all 12 rice chromosomes, with no apparent clustering preference (Figure 2a). The expression levels of all identified these lncRNAs are provided in Supplementary Table S4. The expression levels of lncRNAs were generally lower than those of mRNAs (Figure 2b), and global lncRNA expression levels did not significantly differ among the three treatment groups (Figure 2c).

The expression levels of lncRNAs were first compared between the three treatment groups. A differential expression analysis was then conducted using the DESeq2 package (v1.38.3) in R, with thresholds set at |log_2_(fold change)| > 1 and adjusted q-value < 0.05. The DElncRNAs identified from these comparisons are shown in Figure 2d. A total of 36 DElncRNAs were detected in response to IR56-BPH infestation (IR56-IR vs. IR56-CK), while 46 were identified following TN1-BPH infestation (IR56-TN vs. IR56-CK). Among these, 22 were upregulated in the IR56-BPH group and 35 were upregulated in the TN1-BPH group. To explore population-specific lncRNA responses, we conducted a Venn diagram analysis (Figure 2e). A total of 46 upregulated DElncRNAs were differentially expressed in response BPH infestation (only in IR56-IR vs. IR56-CK and IR56-TN vs. IR56-CK). Eleven DElncRNAs were commonly upregulated under both BPH infestations, while eleven were uniquely upregulated in response to IR56-BPH, and twenty-four were specific to TN1-BPH. In parallel, we identified 729 upregulated differentially expressed mRNAs after TN1-BPH infestation and 487 after IR56-BPH infestation (Figure 2f). These results imply that rice may activate distinct lncRNA-mediated regulatory mechanisms to cope with BPH populations of varying virulence.

### 2.3. Identification of Potential Target Genes of lncRNAs and mRNA

**Figure 3:**
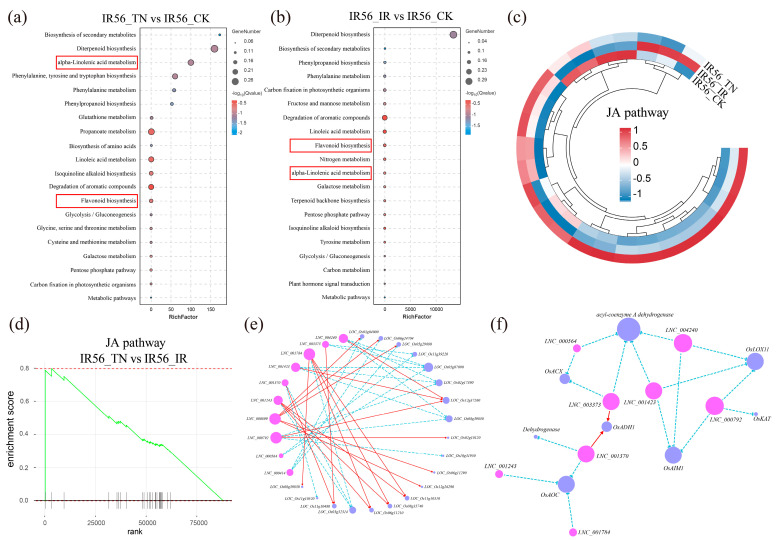
Functional and network analyses of lncRNAs related to the jasmonic acid (JA) pathway. (**a**) KEGG pathway enrichment analysis of differentially upregulated genes after TN1-BPH infestation (IR56-TN vs. IR56-CK). The top 20 significantly enriched pathways are displayed, with “α-Linolenic acid metabolism” and “Flavonoid biosynthesis” highlighted. (**b**) KEGG pathway enrichment analysis of differentially upregulated genes after IR56-BPH infestation (IR56-IR vs. IR56-CK). “α-Linolenic acid metabolism” and “Flavonoid biosynthesis” are also significantly enriched. (**c**) Heatmap showing the expression patterns of genes associated with the JA pathway across the three groups (IR56-CK, IR56-IR, and IR56-TN). Red and blue colors indicate upregulation and downregulation, respectively. (**d**) Gene set enrichment analysis (GSEA) for the JA pathway in the comparison of IR56-TN vs. IR56-IR. (**e**) Co-expression network of lncRNAs and JA-related genes. Correlation analysis identified lncRNA-mRNA interactions with correlation coefficients greater than 0.95 or less than −0.95. Red solid lines suggest potential positive regulatory relationships, while turquoise dashed lines indicate possible negative regulatory interactions. (**f**) Sub-network of lncRNAs regulating key JA-related genes, including *lipoxygenase* (*OsLOX*, *LOC_Os04g37430*), *allene oxide synthase* (*OsAOS*, *LOC_Os03g55800*, *LOC_Os03g12500*), *12-oxophytodienoate reductase* (*OsOPR*, *LOC_Os06g11290, LOC_Os08g35740*, *LOC_Os06g11210*), and *acyl-CoA oxidase* (*OsACX*, *LOC_Os06g24704*). Red solid lines suggest potential positive regulatory relationships, while turquoise dashed lines indicate possible negative regulatory interactions.

A KEGG pathway enrichment analysis was conducted on 1612 upregulated genes following TN1-BPH infestation, and the top 20 enriched pathways are presented in Figure 3a. The expression levels of all mRNAs are provided in Supplementary Table S5. The most significantly enriched KEGG pathways included “Biosynthesis of secondary metabolites”, “Diterpenoid biosynthesis”, “α-linolenic acid metabolism”, “Flavonoid biosynthesis”, and “Glutathione metabolism”. Notably, the “Flavonoid biosynthesis” and “α-linolenic acid metabolism” pathway were also significantly enriched among upregulated genes following IR56-BPH infestation (Figure 3b). Flavonoids are well known for their roles in plant development and stress resistance [20]. α-Linolenic acid serves as precursor for JA and methyl-jasmonate biosynthesis [21]. These findings suggest that the flavonoids and JA signaling pathways may play a critical role in rice responses to BPH infestation.

To further explore the relationship between coding and noncoding regulators of the flavonoid biosynthesis and JA signaling pathways and glutathione metabolism, we performed lncRNA-mRNA coexpression analysis. In the flavonoid biosynthesis pathway, *chalcone synthase* (*OsCHS*, *LOC_Os11g32650*) and *cinnamate-4-hydroxylase* (*OsC4H*, *LOC_Os02g26810*) were significantly upregulated following both IR56-BPH and TN1-BPH infestation [22,23]. Notably, the downstream gene *leucoanthocyanidin reductase* (*OsLAR*, *LOC_Os04g53850*) was specifically downregulated after IR56-BPH infestation [24]. Further analysis revealed that *LNC_002533* expression was positively correlated with that of *OsLAR*. The comparative analysis of JA-related gene expression revealed that infestation by both IR56-BPH and TN1-BPH significantly upregulated JA pathway genes in IR56 rice, with TN1-BPH triggering a more robust induction (Figure 3c). Gene Set Enrichment Analysis (GSEA) further showed significant enrichment of the JA-related gene (normalized enrichment score = 1.49, FDR = 0.15) in IR56-TN compared to IR56-IR (Figure 3d), suggesting the stronger activation of JA-mediated defense responses when IR56 rice is infested by the low-virulence TN1-BPH [25]. In addition, we examined lncRNA-mRNA expression correlation profiles by integrating transcriptomic data. Only lncRNA-mRNA pairs with Pearson correlation coefficients greater than 0.95 or less than −0.95 were retained. This analysis revealed that 10 lncRNAs, specifically upregulated in IR56-IR, were significantly and strong correlations with JA-related genes (Figure 3e). Further analysis indicated that multiple lncRNAs were negatively correlated the expression of key JA-related genes, including the *lipoxygenase* (*OsLOX*, *LOC_Os04g37430*), *allene oxide synthase* (*OsAOS*, *LOC_Os03g12500*, *LOC_Os03g55800*), *12-oxophytodienoate reductase* (*OsOPRs*, *LOC_Os06g11210*, *LOC_Os06g11290*, *LOC_Os08g35740*), and rice *acyl-CoA oxidase* (*OsACX*, *LOC_Os06g24704*) (Figure 3f) [26,27,28,29,30,31]. We also found that *LNC_004240*, *LNC_000792*, and *LNC_001423* were positively correlated with the expression of *OsLOX*, while *LNC_003373* and *LNC_000564* were positively correlated with the expression of *OsACX*. These results suggest that the identified lncRNAs may participate in modulating JA signaling and represent a candidate regulator of IR56 rice susceptibility to IR56-BPH. Glutathione metabolism was specifically enriched only after TN1-BPH infestation (Figure 3a). Further analysis revealed that *LNC_001986* and *LNC_000397* were positively correlated with the expression of *OsGSTL2* (*LOC_Os03g17470*) and *OsGSTF5* (*LOC_Os01g27210*), respectively, in the glutathione metabolism pathway, suggesting that these two lncRNAs may enhance the resistance of IR56 rice to TN1-BPH infestation [32,33].

### 2.4. Validation of the BPH-Feeding Response in IR56-Associated lncRNAs and Their Putative Targets JA Genes

**Figure 4:**
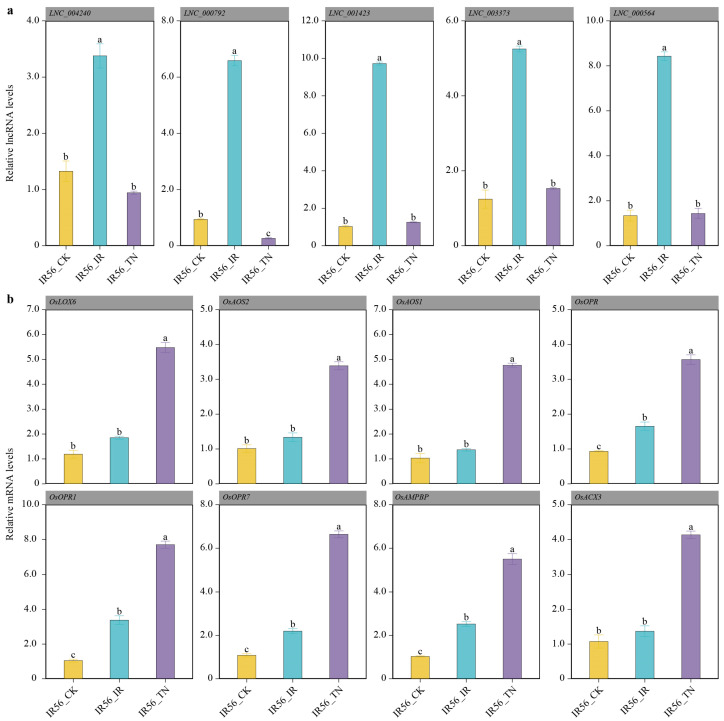
Differential expression of lncRNAs and associated genes in IR56 rice after BPH infestation. (**a**) Relative expression levels of lncRNAs *LNC_004240*, *LNC_000792*, *LNC_001423*, *LNC_003373*, and *LNC_000564* in IR56 rice under control (IR56_CK), IR56-BPH infestation (IR56_IR), and TN1-BPH infestation (IR56_TN) conditions. (**b**) Expression of genes associated with the jasmonic acid (JA) pathway, including *OsLOX6* (*LOC_Os04g37430*), *OsAOS2* (*LOC_Os03g12500*), *OsAOS1* (*LOC_Os03g55800*), *OsOPR* (*LOC_Os06g11210*), *OsOPR1* (*LOC_Os06g11290*), *OsOPR7* (*LOC_Os08g35740*), *OsAMPBP* (*LOC_Os03g04000*), and *OsACX3* (*LOC_Os06g24704*) in IR56 rice after BPH infestation. Different lowercase letters indicate significant differences (*p* < 0.05, Tukey’s test); same letters denote no significant difference. Letters do not reflect difference magnitudes.

To further validate the regulatory involvement of lncRNAs in IR56 rice response to BPH populations with different virulence levels, several DElncRNAs and their putative target genes were selected for qRT-PCR analysis. Five lncRNAs (*LNC_004240*, *LNC_000792*, *LNC_001423*, *LNC_003373*, and *LNC_000564*) showed a markedly higher expression in IR56-BPH-infested IR56 rice plants compared to TN1-BPH-infested and non-infested plants (Figure 4a). In contrast, we observed distinct expression patterns of their target mRNAs differed between IR56-BPH and TN1-BPH infestation (Figure 4b). The expression of *OsLOX6* (*LOC_Os04g37430*), a lipoxygenase gene involved in the early stages of JA biosynthesis, was markedly induced only in IR56 rice after infestation by TN1-BPH populations. Additionally, *OsAOS1* (*LOC_Os03g55800*) and *OsAOS2* (*LOC_Os03g12500*), both allene oxide synthases involved in converting fatty acids to jasmonates, also showed increased expression in response to TN1-BPH infestation. In contrast, IR56-BPH infestation did not significantly alter the expression levels of these genes. *OsOPR* genes, including *OsOPR1* (*LOC_Os06g11290*), *OsOPR7* (*LOC_Os08g35740*), and *OsOPR* (*LOC_Os06g11210*), were significantly upregulated in IR56 rice in response to both IR56-BPH and TN1-BPH infestation. Furthermore, *OsAMPBP* (*LOC_Os03g04000*), a gene associated with antimicrobial peptide biosynthesis, showed moderate upregulation after IR56-BPH infestation and stronger induction following TN1-BPH infestation [34]. This may reflect a differential defense activation in response to BPH virulence. Similarly, *OsACX3* (*LOC_Os06g24704*), an acyl-CoA oxidase gene involved in the fatty acid metabolism pathway, was upregulated in response to IR56-BPH infestation, potentially contributing to the defense-related metabolite production in rice.

## 3. Discussion

### 3.1. Regulation of Rice Defense Against Brown Planthopper by lncRNAs

Although previous studies have examined lncRNAs in rice, the specific roles of these noncoding RNAs in resistance to BPH populations with different virulence levels are still not well understood, highlighting the need for this research. lncRNAs have been identified in various plants, such as *Arabidopsis*, rice, maize, wheat, soybean, tomato, and cotton [10]. These lncRNAs exhibit key characteristics, including tissue-specific expression, low conservation, interaction with other molecules for their function, and alternative splicing, which all point to their crucial roles in plant growth, stress responses, and epigenetic regulation [10]. As one of the most significant pests affecting rice, understanding how lncRNAs regulate resistance to BPH is essential for developing rice varieties with broad-spectrum, durable resistance. However, the exact mechanisms by which rice lncRNAs mediate resistance to BPH, particularly in response to BPH populations with varying virulence, remain unclear.

In this study, we identified a total of 4321 rice lncRNAs, providing valuable insights into functional genomics research in rice, particularly the mechanisms of resistance in rice varieties against BPH infestation. Among the identified lncRNAs, a comparative transcriptomic analysis revealed 60 significantly upregulated lncRNAs in IR56 rice infested by BPH populations with varying virulence levels. Specifically, 22 lncRNAs were upregulated in response to IR56-BPH, while 35 were upregulated following TN1-BPH infestation. Notably, 10 lncRNAs were uniquely upregulated after IR56-BPH infestation, whereas 24 were exclusively upregulated in response to TN1-BPH infestation. These findings underscore the distinct transcriptomic responses elicited by the two BPH populations, reflecting differences in their virulence and the corresponding defense mechanisms activated in the host.

Previous studies have demonstrated that the susceptible rice variety NIP exhibits a greater number of DElncRNAs both before and after BPH infestation compared to the resistant *Bph6* transgenic rice, highlighting the differential regulatory roles of lncRNAs in susceptible versus resistant varieties [35]. Similarly, rice varieties TN1, IR36 (harboring *Bph2*), and R476 (harboring *Bph14* and *Xa21*) were reported to exhibit 84, 52, and 63 specific DElncRNAs, respectively, after BPH infestation [36]. These findings suggest that susceptible rice varieties may rely on a more extensive and diverse set of lncRNAs to mediate their defense responses compared to resistant varieties. In the context of our study, the differential expression of lncRNAs in IR56 rice under infestation by BPH populations with varying virulence levels aligns with these observations. Specifically, the higher number of upregulated lncRNAs in response to TN1-BPH (low virulence) compared to IR56-BPH (high virulence) suggests that IR56 rice may require a more complex lncRNA-mediated regulatory network to counteract low-virulence BPH, whereas high-virulence BPH may suppress or evade certain defense mechanisms, leading to fewer lncRNA responses. Moreover, the lncRNAs uniquely expressed in response to varying BPH virulence levels are likely pivotal in the loss of resistance IR56 rice to high-virulence brown planthoppers (IR56-BPH).

### 3.2. Regulation of Secondary Metabolite Synthesis in Rice by lncRNAs

lncRNAs are critical regulators of biological processes, with numerous studies demonstrating their role in modulating mRNA expression [37,38,39]. This study revealed that differentially upregulated genes following feeding by two BPH populations were significantly enriched in the flavonoid biosynthesis pathway, with greater enrichment observed after TN1-BPH infestation. Specifically, after IR56-BPH infestation, *LNC_002533* significantly suppressed the expression of *leucoanthocyanidin reductase* (*OsLAR, LOC_Os04g53850*) within this pathway, suggesting that lncRNAs may reduce rice resistance to BPH by modulating flavonoid (e.g., proanthocyanidin) biosynthesis. Flavonoids are well-established as protective compounds in plants, offering defense against various pathogens and insects [40]. Previous research has shown that increased flavonoid content enhances rice resistance to BPH, with a novel resistance mechanism mediated by the OsmiR396-OsGRF8-OsF3H-flavonoid pathway [41]. This study extends the understanding of lncRNAs in regulating flavonoid biosynthesis, highlighting their potential role in rice defense mechanisms against BPH. However, further experimental validation, such as functional studies using CRISPR/Cas9 or RNAi approaches, is needed to confirm the specific regulatory roles of lncRNAs like *LNC_002533* in flavonoid synthesis and their impact on BPH resistance.

Glutathione is known to enhance rice stress resistance, as demonstrated in previous studies [42,43]. It has been reported that glutathione metabolism plays a critical role in rice resistance to pests, and differential lncRNAs mediate the adaptability of BPH to resistant and susceptible rice varieties, with glutathione metabolism being a key component of this process [44]. Furthermore, studies have demonstrated that the expression of miRNAs and lncRNAs is associated with glutathione metabolism in rice under stress conditions. For example, in salt-sensitive and salt-tolerant rice varieties, the expression of miR365f increases with prolonged salt treatment, and this miRNA is closely associated with glutathione metabolism, modulating oxidative stress responses and thereby enhancing salt tolerance [45]. Similarly, lncRNAs have been implicated in the regulation of glutathione-related pathways, influencing plant defense mechanisms against abiotic stresses [46]. This study reveals that *LNC_001986* and *LNC_000397* may directly or indirectly modulate glutathione metabolism, thereby activating defense mechanisms against low-virulence BPH infestations. These lncRNAs may act as key regulators in enhancing IR56 rice resistance to TN1-BPH.

Based on these findings, we propose that after IR56-BPH infestation, *LNC_002533* suppresses flavonoid (e.g., proanthocyanidin) biosynthesis, thereby reducing the resistance of IR56 rice to IR56-BPH. In contrast, after TN1-BPH infestation, *LNC_001986* and *LNC_000397* upregulate the glutathione metabolism pathway, enhancing the resistance of IR56 rice to TN1-BPH. These results provide important mechanistic insights into how lncRNAs regulate defense pathways and offer potential targets for the genetic enhancement of rice resistance to BPH.

### 3.3. lncRNAs Regulate the Jasmonic Acid Pathway in Rice After Brown Planthopper Infestation

Plant hormones, JA and salicylic acid, are critical regulators of rice defense responses against pests and diseases. Numerous studies have explored the role of the JA signaling pathway in modulating rice resistance to BPH infestations [47,48]. Our study identified a set of rice lncRNAs that regulate the signal transduction of the JA pathway, suggesting that lncRNAs may influence JA synthesis to enhance defense against BPH.

Alpha-linolenic acid, a key precursor for JA synthesis, plays a pivotal role in determining JA accumulation in plants [21]. Research has shown that the production of alpha-linolenic acid is a rate-limiting step for the synthesis of JA and JA-Ile in response to injury in rice [49]. In this study, we observed that IR56 rice infested by IR56-BPH exhibited an increased expression of lncRNAs such as *LNC_004240*, *LNC_000792*, and *LNC_001423*. These lncRNAs may inhibited the enzymatic conversion of alpha-linolenic acid catalyzed by *OsLOX*, an essential step in the JA biosynthesis pathway. Additionally, downstream in the JA pathway, *LNC_003373* and *LNC_000564* further suppressed the expression of *OsACX*, ultimately reducing JA content in IR56 rice plants infested by IR56-BPH. In contrast, the JA pathway in IR56 rice plants infested by TN1-BPH remained unaffected by lncRNAs, preserving JA biosynthesis. Supporting evidence from bacterial blight infection studies in rice revealed that GO and KEGG analyses of lncRNAs responsive to *Xoo* highlighted enrichment in the alpha-linolenic acid metabolic pathway [15]. For instance, the lncRNA *ALEX1* promotes alpha-linolenic acid metabolism by upregulating key genes such as *OsAOC*, *OsOPR7*, and *OsJAR1*, leading to increased JA accumulation and enhanced rice resistance to *Xoo* [15].

**Figure 5:**
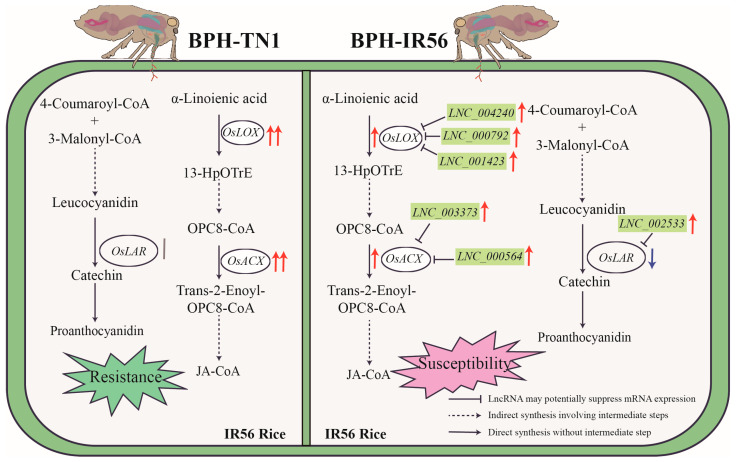
Differential regulation of the jasmonic acid (JA) pathway in IR56 rice infested by TN1-BPH and IR56-BPH. This schematic illustrates the contrasting regulatory roles of lncRNAs in the JA biosynthesis pathway and flavonoid (e.g., proanthocyanidin) biosynthesis under infestations by TN1-BPH (left) and IR56-BPH (right) in IR56 rice. TN1-BPH infestation (resistance): Under TN1-BPH infestation, α-linolenic acid is metabolized into JA derivatives through the sequential actions of *OsLOX* (*LOC_Os04g37430*) and *OsACX* (*LOC_Os06g24704*), which are upregulated. The upregulation of these JA pathway genes enhances JA-CoA synthesis. These factors contributed to enhanced resistance of IR56 rice to TN1-BPH. IR56-BPH infestation (susceptibility): In contrast, IR56 rice infested by IR56-BPH shows reduced partial JA biosynthesis. Specific lncRNAs, such as *LNC_004240*, *LNC_000792*, and *LNC_001423*, may suppress the expression of the JA biosynthesis gene *OsLOX* (*LOC_Os04g37430*), while *LNC_003373* and *LNC_000564* may inhibit *OsACX* (*LOC_Os06g24704*) expression, resulting in decreased JA-CoA production and weakened defense responses. Concurrently, the expression of the key enzyme *OsLAR* (*LOC_Os04g53850*) in the phenylalanine metabolic pathway was downregulated by *LNC_002533*, potentially reducing proanthocyanidin production. These metabolic changes may collectively contribute to the increased susceptibility of IR56 rice to IR56-BPH. Visual elements are represented as follows: red upward arrows for upregulation, blue downward arrows for downregulation, gray vertical bars for no significant change, and multiple arrows indicate greater fold changes in expression.

While our findings align with previous studies demonstrating the regulatory roles of lncRNAs in JA biosynthesis, there are notable differences in the mechanisms observed. For example, ALEX1 enhances JA accumulation by upregulating JA biosynthesis genes, whereas the lncRNAs identified in this study, including *LNC_004240*, *LNC_000792*, *LNC_001423*, *LNC_003373*, and *LNC_000564*, appear to suppress JA biosynthesis, rendering IR56 rice more susceptible to high-virulence BPH. Based on these findings, we hypothesize that the identified lncRNAs reduce JA biosynthesis in IR56 rice by suppressing key steps in the JA pathway, thereby diminishing resistance to high-virulence BPH infestations (Figure 5). Despite these insights, our study has limitations. For instance, the exact mechanisms by which these lncRNAs regulate *OsLOX* and *OsACX* expression remain unclear. Future studies should employ CRISPR/Cas9-based knockout or the overexpression of these lncRNAs to validate their roles in JA biosynthesis and BPH resistance.

## 4. Materials and Methods

### 4.1. Plant Materials and Growth Conditions

The rice variety IR56 is an indica rice cultivar that harbors the BPH resistance gene *Bph3*. Germinated IR56 seeds were sown in seedling trays within a screen house under standard light, temperature, and humidity conditions. Fourteen-day-old seedlings were transferred to a greenhouse at the China National Rice Research Institute, where environmental conditions were maintained at 28 ± 2 °C, 75-85% relative humidity, and a photoperiod of 16 h of light and 8 h of darkness.

### 4.2. Insect Materials and Growth Conditions

The brown planthopper (BPH) population was originally collected from paddy fields in Hangzhou, China. This population has been maintained at the China National Rice Research Institute (CNRRI) on TN1 rice (TN1-BPH) and IR56 rice (IR56-BPH) under the previously described conditions for over 12 years [50]. The virulence levels of TN1-BPH and IR56-BPH differ significantly [51].

### 4.3. Sample Collections and RNA Isolation

Fourteen-day-old IR56 seedlings were transferred to plastic cages (10 cm in diameter, 60 cm in height) equipped with a 0.05 mm pore-size mesh. Four newly emerged adult female BPHs were released into each cage, which was then covered with transparent gauze and incubated in a greenhouse under controlled conditions: 28 ± 2 °C, 75–85% relative humidity, and a photoperiod of 16 h of light and 8 h of darkness. IR56 seedlings not exposed to BPHs were placed in similar plastic cages and served as the IR-CK control group. Each experimental treatment included three biological replicates, with three plants per replicate and four BPH individuals per plant. Plant stems from both the control and treatment groups were collected 24 h after BPH infestation, immediately frozen in liquid nitrogen, and stored at −80 °C for subsequent analysis. TransZol Up (Transgen, Beijing, China) was used to isolate total RNA from each sample (no BPH, IR56-CK; IR56-BPH infested, IR56-IR; TN1-BPH infested, IR56-TN).

### 4.4. Library Construction and Sequencing

First, ribosomal RNA was removed from the integrated and qualified RNA samples, after which the RNA was fragmented using divalent cations under elevated temperatures. The resulting fragmented RNA was then used to construct strand-specific cDNA libraries according to standard Illumina protocols, and sequencing was carried out on an Illumina HiSeq 2500 platform (Illumina, San Diego, CA, USA). The raw sequencing reads were subjected to several rigorous filtering steps to ensure the quality of the data for subsequent analysis. Initially, adapter-containing reads were removed to prevent interference from sequencing artifacts. Next, reads with a high proportion of ambiguous bases (N), specifically those with more than 10% N, were excluded. Lastly, low-quality reads were filtered out based on predefined quality thresholds. The remaining high-quality reads, referred to as clean reads, were used for downstream analyses.

### 4.5. Identification of lncRNAs

The clean reads were aligned to the rice genome using the HISAT2 (v2.1.0) software [52]. The mapped reads were subsequently assembled with StringTie (v1.3.5) [53]. The assembled transcripts were annotated using the gffcompare (v0.12.6) program to identify protein-coding mRNAs. Unknown transcripts longer than 200 nucleotides and containing at least one exon were selected as lncRNA candidates. The final lncRNAs were obtained by filtering the putative protein-coding RNAs through three computational approaches: CPC2 (CPC score > 0) [54], CNCI (CNCI score > 0), and Pfam [55,56].

### 4.6. Differential Expression Analyses of mRNAs and lncRNAs

To compare the expression of lncRNAs and mRNAs across different virulence levels (IR56-CK, IR56-IR, and IR56-TN), the expression levels were quantified using FPKM (fragments per kilobase per million mapped fragments) with the StringTie software. Gene expression differences between treatment groups were analyzed using the DESeq2 package with read count data in R [57]. Differentially expressed genes (DEGs) and lncRNAs (DELs) were identified based on a statistical significance threshold of q-value < 0.05 and an absolute log2(fold change) > 1. Gene locus identifiers and annotations were retrieved from the MSU Rice Genome Annotation Project (Release 7).

### 4.7. KEGG Pathway Analysis and Network Construction

Potential target genes of lncRNAs were predicted based on their regulatory patterns, including both cis- and trans-acting mechanisms. Cis-target genes were identified by locating protein-coding genes colocalized within 10 kb upstream or downstream of individual lncRNAs. To classify these putative cis-target genes, KEGG pathway analyses were performed using the clusterProfiler (v4.10.0) R package [58].

Rice genes involved in JA biosynthesis, along with their annotations, were retrieved from the literature, and their interactions with lncRNAs were analyzed [34]. Gene Set Enrichment Analysis was performed using the GSEA (v4.2.3) program with screening criteria of FDR q-value < 0.2, and the JA-related gene set was curated from published defense response studies [25,34]. mRNAs with correlation coefficients greater than 0.95 or less than −0.95 were identified as potential lncRNA targets. The lncRNA-mRNA interactions were then visualized as a network using the Cytoscape (v3.9.0) software.

### 4.8. cDNA Synthesis

Before analysis, the rice samples were placed in 1.5 mL centrifuge tubes containing 2 mm zirconium oxide grinding beads (Servicebio, Wuhan, China), immediately frozen with liquid nitrogen, and ground into powder using a tissue grinder (LICHEN, Shanghai, China) at 70 Hz for 120 s. Total RNA was extracted using TransZol Up reagent (Transgen, Beijing, China), following the manufacturer’s protocol. The RNA concentration and quality were assessed using a NanoDrop ND-1000 spectrophotometer (Thermo Fisher Scientific, Waltham, MA, USA) and, if needed, by 1% (*w*/*v*) agarose gel electrophoresis. cDNA was synthesized using ReverTra Ace qPCR RT Master Mix with gDNA Remover (Toyobo, Osaka, Japan), as per the manufacturer’s instructions.

### 4.9. Quantitative Real-Time PCR (qPCR) and Statistical Analysis

Primers for all lncRNAs and defense-related genes were designed using the Sangon Biotec primer design webpage (https://store.sangon.com/newPrimerDesign, accessed on 24 November 2024). According to the manufacturer’s instructions, a 10 µL reaction mixture was prepared using SYBR Green Realtime PCR Master Mix (Toyobo, Osaka, Japan), and qPCR was performed on an ABI 7500 real-time PCR system (Applied Biosystems, Foster City, CA, USA). Each reaction included three independent biological samples, with each biological sample having three valid technical replicates. The constitutively expressed housekeeping gene *OsUbq* (*LOC_Os01g22490*) from rice was used as an endogenous control [59]. Relative expression was calculated using the 2^−∆∆Ct^ method [60]. All primers used in the study are listed in Supplemental Table S6.

The statistical analysis of the relative expressions was performed using R. All values are presented as mean ± SE. After respectively testing for normality (Shapiro-Wilk test) and homogeneity variance (Levene’s tests), these data were further evaluated by a one-way analysis of variance following by Tukey’s honestly significant difference test to determine the differences across various treatments. The difference was considered as significant or extremely significant when the *p*-value below 0.05.

## 5. Conclusions

This study provides a comprehensive characterization of lncRNAs in rice and their regulatory roles in responses to BPH infestations with varying virulence levels. A total of 4321 lncRNAs were identified, with 60 significantly upregulated lncRNAs observed under BPH stress. Through transcriptome analysis and qPCR validation, we demonstrated that lncRNAs modulate critical defense pathways, including JA signaling and flavonoid biosynthesis, highlighting their potential roles as upstream regulators of these processes. Notably, lncRNAs such as *LNC_004240*, *LNC_003373*, *LNC_002533*, *LNC_001986* and *LNC_000397* were implicated in the regulation of JA, flavonoid biosynthesis, and glutathione metabolism, offering mechanistic insights into rice–BPH interactions. Our findings underscore the potential of lncRNAs as molecular targets for improving rice resistance to insect herbivory. To further validate their functional relevance, future work should include CRISPR/Cas9-mediated knockout or the overexpression of candidate lncRNAs (e.g., *LNC_002533* and *LNC_001986*) to confirm their roles in BPH resistance and defense regulation.

## Data Availability

Data are contained within the article and Supplementary Materials.

## Supplementary Materials

The following supporting information can be downloaded at: https://www.mdpi.com/article/10.3390/ijms26083486/s1.

## Abbreviations

The following abbreviations are used in this manuscript:

BPH	Brown planthopper
lncRNAs	Long noncoding RNAs
DElncRNAs	Differentially expressed lncRNAs
JA	Jasmonic acid
IR56-BPH	Brown planthopper population reared on IR56 rice
TN1-BPH	Brown planthopper population reared on TN1 rice
IR56-CK	IR56 rice without brown planthopper infestation
IR56-IR	IR56 rice without IR56-BPH infestation
IR56-TN	IR56 rice without TN1-BPH infestation
